# Kawasaki Disease in Mongolia: Results From 2 Nationwide Retrospective Surveys, 1996–2008

**DOI:** 10.2188/jea.JE20100144

**Published:** 2011-07-05

**Authors:** Dambadarjaa Davaalkham, Yosikazu Nakamura, Davaakhuu Baigalmaa, Gombojav Davaa, Ochir Chimedsuren, Nyamjav Sumberzul, Tserenkhuu Lkhagvasuren, Ritei Uehara, Hiroshi Yanagawa, Tomisaku Kawasaki

**Affiliations:** 1 Department of Epidemiology and Biostatistics, School of Public Health, Health Sciences University of Mongolia, Ulaanbaatar, Mongolia; 2 Department of Public Health, Jichi Medical University, Shimotsuke, Japan; 3 Department of Surveillance and Response, National Center for Communicable Diseases, Ulaanbaatar, Mongolia; 4 Health Sciences University of Mongolia, Ulaanbaatar, Mongolia; 5 Japanese Kawasaki Disease Research Center, Tokyo, Japan

**Keywords:** mucocutaneous lymph node syndrome, nationwide survey, Mongolia, children

## Abstract

**Background:**

Kawasaki disease (KD) has been reported in many countries. However, the incidence of KD in Mongolia is not known. This is the first report of incident cases of KD in Mongolia, which were identified using data from 2 nationwide surveys.

**Methods:**

Two nationwide retrospective surveys were conducted: medical histories were collected from patients aged 0 to 16 years who were hospitalized countrywide between 1996 and 2008. Hospital records for these patients were also reviewed. Nationwide training seminars on KD were conducted before each survey.

**Results:**

For the nationwide surveys, the participation rates among all hospitals with pediatric wards were 97% and 94%. Inpatient medical histories from 1996 through 2008 were reviewed, and, among children younger than 16 years, 9 patients with KD were investigated. The age of KD patients ranged from 1.4 to 14 years; 7 of 9 patients were male. Six (67%) patients fulfilled all 6 clinical diagnostic criteria; the other 3 (33%) were defined as having KD based on the presence of 5 such criteria. Fever persisting 5 or more days, bilateral conjunctival congestion, and changes of the lips and oral cavity were the most common symptoms, and cervical lymphadenopathy was the least common symptom. Cardiac sequelae developed in 5 of the patients, 4 of whom were older than 10 years.

**Conclusions:**

The results of these nationwide surveys reveal that KD cases do exist in Mongolia. However, knowledge of KD among Mongolian pediatricians is likely to be poor. Thus, there is a need to augment their understanding to improve management of KD patients. Further studies are crucial to clarify the epidemiologic characteristics of KD in Mongolia.

## INTRODUCTION

Kawasaki disease (KD; also known as acute infantile febrile mucocutaneous lymph node syndrome) was originally described as a distinct clinical entity in Japanese children by Dr. Tomisaku Kawasaki in 1967^1^ and was first mentioned in the English literature in 1974.^2^ KD is now recognized as the leading cause of acquired heart disease among children in developed countries. It is an acute self-limited vasculitis of infancy and early childhood and is characterized by fever, bilateral nonexudative conjunctivitis, erythema of the lips and oral mucosa, changes in the extremities (ie, reddening of palms and soles, indurative edema, and membranous desquamation), rash, and cervical lymphadenopathy. Although an infectious agent is suspected, the exact cause is unknown. However, considerable progress has been made in understanding the natural history of KD, and therapeutic interventions that reduce the immune-mediated destruction of the arterial wall have been developed.^3^ Coronary artery aneurysms or ectasia develop in approximately 20% to 30% of untreated children and can lead to ischemic heart disease or sudden death.^4^

KD patients have been identified in more than 60 countries, and KD has become the leading cause of acquired heart disease among children in developed countries. The reported incidence of KD varies widely by country. Incidence is highest in Japan, and the numbers continue to rise, possibly due to heightened awareness of the condition. Recent reports from Japan show that the incidence of KD increased from 184.6 to 216.9 per 100 000 children aged 0 to 4 years from 2007 to 2008.^5^^,^^6^

The rate of KD in South Korean children younger than 5 years is estimated at 86.4/100 000, while the reported incidence in Taiwan is 66/100 000.^7^^,^^8^ The highest KD incidence in the United States was in Hawaii, 17.1/100 000.^9^ The overall incidence of KD in the United Kingdom was 8.1/100 000; however, it was reported to be 14.6/100 000 among children of parents from the Indian subcontinent.^10^

Located in the northern part of central Asia, Mongolia is a landlocked country bordered by Russia to the north and China to the south. With a total area of 1.6 million km^2^ and a population of 2.6 million as of 2008 (1.5 inhabitants per km^2^), Mongolia is one of the most sparsely populated countries in the world. In 2002, China reported a KD incidence of 36.8/100 000 children younger than 5 years.^11^ There has been no systematic study of KD in Mongolia. Therefore, we conducted nationwide surveys of KD incidence in Mongolia in 2005 and 2008.

## METHODS

### Study design and setting

We conducted a retrospective survey to investigate KD incidence among inpatients in Mongolia. Administratively, Mongolia is divided into 21 provinces; the capital, Ulaanbaatar city, comprises 9 districts. A total of 32 hospitals were included in the survey. This included the General Provincial Hospitals of all the provinces, the District Health Centers for all districts in Ulaanbaatar, the National Center for Communicable Diseases, and the Maternal and Children’s Research Center. These hospitals represent all pediatric wards in Mongolia in which children younger than 16 years are hospitalized.

### Nationwide training seminar on KD

In collaboration with the Japan Kawasaki Disease Research Center (Tokyo 101-0041, Japan, Professor Tomisaku Kawasaki), we organized training seminars on KD diagnosis and treatment and our research methodology for pediatricians working throughout the country—including urban and rural areas. The training sessions were held in September in 2005 and 2008, ie, before the 2 national surveys, to provide information to pediatricians on the diagnosis and management of KD and standardized data collection procedures. Head physicians of the pediatric wards were invited to Ulaanbaatar for the training sessions. The cumulative participation rates were 94% and 100% in 2005 and 2008, respectively. The second seminar included pediatricians who had not attended the first seminar. An interview-based survey was used to assess the pediatrician’s knowledge of KD before training. The results of this pre-training survey revealed that almost 97% of Mongolian pediatricians had a poor understanding of KD. After each seminar, these newly trained pediatricians organized training for local doctors in their hospitals (“train the trainer”).

### Data collection

After the first seminar, a nationwide survey of KD was initiated in collaboration with trained pediatricians. The medical histories of patients aged 0 to 16 years who were hospitalized during the period from 1996 through 2005 were retrieved in the first survey. The medical records of those who were hospitalized during the period from 2006 through 2008 were retrieved in the second survey to identify cases that satisfied the diagnostic guidelines of KD. The latest (5th) version of the “Diagnostic Guidelines of Kawasaki Disease” was translated into Mongolian and distributed to all hospitals, along with color photographs of the clinical features of KD. A classical diagnosis of KD is based on the presence of both a fever persisting at least 5 days and at least 4 of 5 other principal clinical features (Table 1). These principal clinical symptoms are bilateral conjunctival injection, changes in the lips and oral cavity, changes in the extremities, erythematous rash, and cervical lymphadenopathy. Our methodology accounted for the fact that, in general, not all clinical features of KD are present at a single time point and that a period of observation may thus be necessary before the diagnostic criteria are met. Patients with fever for at least 5 days and 4 of the other 5 principal clinical features received a diagnosis of KD. The presence of coronary artery disease was investigated by using 2-dimensional echocardiography or coronary angiography.

**Table 1.** Diagnostic criteria of Kawasaki disease

Fever persisting at least 5 days
Presence of at least 5 principal features:
Changes in extremities
Acute—Erythema of palms, soles; ​ edema of hands, feet
Subacute—Periungual peeling of fingers, ​ toes in weeks 2 and 3
Polymorphous exanthem
Bilateral bulbar conjunctival injection without exudate
Changes in lips and oral cavity—Erythema, ​ lips cracking, strawberry tongue, ​ diffuse injection of oral and pharyngeal mucosae
Cervical lymphadenopathy (>1.5 cm in diameter), usually unilateral

A brief survey questionnaire was provided to obtain information on KD patients and the details of their hospitalization. The questionnaire asked about demographics, KD symptoms, and cardiac complications.

The study protocols of the surveys were approved by the Institutional Review Board of the Health Sciences University of Mongolia.

## RESULTS

Among all (*n* = 32) hospitals with pediatric wards, the participation rates in the 2 nationwide surveys were 97% and 94% in 2005 and 2008. In these surveys, a total of 241 705 inpatient medical histories were reviewed to identify cases that satisfied the diagnostic criteria for KD. Nearly 200 000 children were hospitalized in the general hospitals of rural provinces; the remaining children were hospitalized in the hospitals of Ulaanbaatar. By means of these surveys, we identified 9 patients aged 0 to 16 years who met the KD case definition. Of these, 4 were observed in the provinces of Dornod, Zavkhan, Uvurkhangai, and Tuv; 4 were hospitalized in Ulaanbaatar (3 in the Maternal and Children’s Research Center and 1 in Chingeltei District Health Center), and 1 case of KD that was not diagnosed in the hospitals of Ulaanbaatar. That patient received a KD diagnosis and was treated in Beijing, China.

As shown in Table 2, the age of our KD patients ranged from 1 year 4 months to 14 years, with a mean (± SD) of 8.4 ± 4.7 years. Seven (78%) of the 9 children were boys. Regarding place of residence, 4 (44%) were living in districts of Ulaanbaatar, and 5 resided in rural provinces. Five (56%) were hospitalized in winter and spring, and the others were hospitalized in summer and autumn.

**Table 2.** Age, sex, residence, and hospital of admission of observed KD patients

Patient No.	Age (years)	Sex	Year and month of onset	Residence	Hospital	Diagnosis^a^	Treatment		Cardiac sequellae

							IVIG^b^	Aspirin	
1	10	Girl	January, 1998	Zavkhan province	General Provincial Hospital	Kawasaki disease	no	no	no
2	13	Boy	September, 2005	Tuv province	General Provincial Hospital	Cervical Lymphadenitis in left side	no	no	no
3	10	Boy	March, 2002	Sukhbaatar district, Ulaanbaatar	District Health Center	Toxico-allergic reaction of unknown ethiology and dermatitis	no	no	no
4	5	Boy	February, 2005	Uvurkhangai province	General Provincial Hospital	Lupus erithrematosis	no	no	yes
5	1.4	Boy	November, 2007	Dornogovi province	General Provincial Hospital	Viral infection	no	no	no
6	14	Boy	March, 2000	Baganuur district, Ulaanbaatar	Maternal and Children’s Research Center	Toxico-allergic reaction of unknown ethiology and dermatitis	no	no	yes
7	10	Boy	June, 2005	Tuv province	Maternal and Children’s Research Center	Kawasaki disease, acute stage	yes	yes	yes
8	11	Boy	November, 2008	Songinokhairkhan district, Ulaanbaatar	Maternal and Children’s Research Center	Kawasaki disease, acute stage	yes	yes	yes
9	1.6	Girl	October, 2009	Bayanzurkh district, Ulaanbaatar	United Family Hospitals and Clinics, Beijing	Kawasaki disease	yes	yes	yes

^a^Diagnosed during hospital admission.

^b^Intravenous immunoglobulin.

There were no deaths.

Six (67%) patients fulfilled all 6 clinical criteria for KD; 2 (22%) patients had persistent fever plus 4 additional clinical KD criteria. Clinical symptoms of changes in the peripheral extremities and polymorphous exanthema were not found in the ninth patient, who was diagnosed in Beijing. Fever persisting 5 or more days, bilateral conjunctival congestion, and changes in lips and oral cavity were the most common symptoms and were observed in all patients. Cervical lymphadenopathy was the least common symptom and was found in 78% of KD patients in Mongolia.

Table 2 also shows diagnosis, treatment, cardiac sequelae, and outcome of children with KD. KD was correctly diagnosed in hospitals in 4 patients: 3 at the Maternal and Children’s Research Center—the largest children’s hospital in Mongolia—and 1 at a hospital in Zavkhan province. The condition of the remaining 5 cases (56%) was misdiagnosed as toxic-allergic reaction of unknown etiology, dermatitis, lupus erythematosus, or viral infection. The standard treatment for KD, namely, intravenous immunoglobulin (IVIG) and aspirin, was administered to only 3 (33%) children. Cardiac sequelae were documented in 56% of children who underwent echocardiography in hospitals in Ulaanbaatar. Unfortunately, echocardiography was not performed for the other patients because it was not available in rural hospitals. According to the medical records, no deaths occurred during hospitalization among the patients.

## DISCUSSION

Before this study and educational undertaking for pediatricians, KD was unknown or under-identified as a disease entity in Mongolia. We conducted these retrospective surveys to determine (1) if KD was present, but undiagnosed, in Mongolia, (2) if Mongolian pediatricians had identified KD, but not reported it, or (3) if KD was not present in the Mongolian population.

In this survey, we abstracted medical records from all hospitals in which children are hospitalized in Mongolia. All other medical facilities in Mongolia have only outpatient pediatric clinics. Therefore, the results of these surveys may represent only those cases in which children with KD were hospitalized and thus perhaps not the entire incidence of KD in Mongolia. The survey results reveal that KD is present in Mongolia. Only a few pediatricians staffing the largest children’s hospital in the capital city were aware of this disease and were able to diagnose it or identify late-stage complications. Additionally, most pediatricians (97%) throughout the country had never heard of KD, which could lead to underdiagnosis of this disease. Before our educational activities, KD had not been part of the educational curriculum of the medical colleges. In collaboration with the Japan Kawasaki Disease Research Committee, we organized national training seminars on KD diagnosis, treatment, and epidemiology for pediatricians in 2005 and 2008. Pediatricians who were trained at those seminars later organized training for KD awareness in their own hospitals. As a result of these efforts, we expect that that diagnosis and treatment of KD patients has improved in Mongolia.

KD is most common in Japan, where the incidence rate is 10 times that in the United States and 30 times that in the United Kingdom and Australia. Worldwide, the annual reported incidence varies from 3.4/100 000 to 174/100 000 children younger than 5 years. In the United States, there were an estimated 4248 hospitalizations for KD among children aged 2 years in 2000.^7^

In our survey, we identified only 9 patients who satisfied the diagnostic criteria for KD among children aged 0 to 16 years who had been hospitalized between 1996 and 2008 in Mongolia. This indicates that although uncommon, KD cases do exist in Mongolia. The small number of observed incident cases could be due to insufficient information on clinical symptoms in the medical records of patients. The pediatricians who participated in these surveys reported that the presence or absence of some symptoms had not been described in the medical records. In addition, echocardiography was available in large hospitals in Ulaanbaatar, but not in rural hospitals, so it was not always performed, even in late stages of the disease. Therefore, it is likely that late-stage complications of KD were not always identified. Alternatively, it may be that KD is very rare in Mongolia. The incidence of KD is generally higher in developed countries. Mongolia is a developing country with a low population density and may thus have a low incidence of KD. Future nationwide epidemiologic surveys will be necessary to determine the incidence of KD in Mongolia. We did observe a higher reported incidence of KD in the second survey between our survey years. This may represent better diagnostic attention and capacity or a true increase in incidence as the population increasingly clusters in Ulaanbaatar. We expect additional cases of KD as awareness and urbanization increase.

Children aged 6 months to 5 years are the most susceptible to KD, with peak incidence occurring in those aged 9 to 11 months.^13^ The ratio of boys to girls is 3:2, and 85% of patients are younger than 5 years.^14^ KD is uncommon in patients 9 years or older^15^^–^^18^: an epidemiologic survey from Japan found that only 1% of cases were children 9 years or older.^18^ In our survey, the age of KD patients ranged from 1.4 to 14 years, and most were boys. Children with KD in Mongolia are older than those in Japan. However, recent reports have emphasized the occurrence of KD in older children, who may have a higher prevalence of cardiovascular complications related to late diagnosis.^19^^–^^21^

Older age at onset of illness may be an independent risk factor for the development of coronary aneurysms in KD,^22^ and cardiac sequelae were observed in more than half of the present patients. This high proportion of cardiac sequelae could be related to the older age of our children at KD diagnosis and/or the lack of available treatment. Three children who received aspirin and IVIG developed coronary abnormalities, possibly due to delayed treatment. It should be noted that 97% of pediatricians countrywide were unaware of KD (only 1 pediatrician, from Ulaanbaatar, had knowledge of KD). Thus, IVIG therapy was not administered and echocardiography was not performed in suspected cases. In addition, it is possible that patients with mild or moderate KD symptoms were younger and were not referred to hospitals for admission due to lack of knowledge among physicians. Therefore, the present surveys might have missed many young patients with KD.

Aspirin and IVIG has been the main therapeutic regimen for KD for many years. Randomized, prospective, clinical trials in the United States in the 1980s established that IVIG was effective and safe, and that it reduced the rate of coronary artery lesions.^23^ Ideally, IVIG should be initiated within the first 10 days of illness.^24^ Previously, the proportion of patients with cardiac sequelae was much higher (>20% until 1986) among patients younger than 1 year, as compared with other age groups. Since 1986, however, the incidence of coronary sequelae has been declining in this age group.

An infectious agent is strongly suspected as the cause of KD in view of both clinical observations and a number of epidemiologic features. Analysis of the seasonal distribution of cases shows peak incidence in late winter/early spring.^25^ Thus, it seems likely that KD is associated with some widely distributed infectious agent.^26^ Nevertheless, no excess hospitalization for KD during winter and spring was found in Mongolia.

In this survey, we observed that fever persisting 5 days or longer, bilateral conjunctival congestion, and changes in lips and the oral cavity were the most common symptoms. Cervical lymphadenopathy was the least common among KD patients in Mongolia. Cervical lymphadenopathy was also identified as the least common of the principal clinical features in other studies.^4^^,^^13^

A limitation of this study was that the surveys were based on a review of medical histories of hospitalized children, ie, there was no follow-up of children with KD. In addition, young children with milder KD might have been underreported in this study because they were unlikely to have been hospitalized.

Nonetheless, this is the first report to identify KD patients in Mongolia, based on data from 2 nationwide retrospective surveys. In these surveys, all urban and rural hospitals with pediatric inpatient wards were included, and the medical history of every child younger than 16 years was reviewed to identify KD patients who were hospitalized throughout the country between 1996 and 2008.

In conclusion, the results of 2 nationwide surveys revealed that KD is present in Mongolia. Knowledge of KD among Mongolian pediatricians should be enhanced to increase diagnostic sensitivity and guarantee appropriate treatment of KD. In addition, further epidemiologic surveys are necessary to clarify the incidence and characteristics of KD in Mongolia.
